# Design and Fabrication of Embroidered Textile Strain Sensors: An Alternative to Stitch-Based Strain Sensors

**DOI:** 10.3390/s23031503

**Published:** 2023-01-29

**Authors:** Jose Guillermo Colli Alfaro, Ana Luisa Trejos

**Affiliations:** 1 School of Biomedical Engineering, Western University, London, ON N6A 5B9, Canada; 2 Department of Electrical and Computer Engineering, Western University, London, ON N6A 5B9, Canada

**Keywords:** textile-based strain sensor, machine-embroidered strain sensor, resistive textile strain sensor, conductive thread

## Abstract

Smart textile sensors have been gaining popularity as alternative methods for the continuous monitoring of human motion. Multiple methods of fabrication for these textile sensors have been proposed, but the simpler ones include stitching or embroidering the conductive thread onto an elastic fabric to create a strain sensor. Although multiple studies have demonstrated the efficacy of textile sensors using the stitching technique, there is almost little to no information regarding the fabrication of textile strain sensors using the embroidery method. In this paper, a design guide for the fabrication of an embroidered resistive textile strain sensor is presented. All of the required design steps are explained, as well as the different embroidery design parameters and their optimal values. Finally, three embroidered textile strain sensors were created using these design steps. These sensors are based on the principle of superposition and were fabricated using a stainless-steel conductive thread embroidered onto a polyester–rubber elastic knit structure. The three sensors demonstrated an average gauge factor of 1.88±0.51 over a 26% working range, low hysteresis (8.54±2.66%), and good repeatability after being pre-stretched over a certain number of stretching cycles.

## 1. Introduction

Continuous monitoring of human motion has been shifting towards the use of smart textile sensors. This is especially important in the context of musculoskeletal rehabilitation, as textile strain sensors have the advantage of being able to be seamlessly integrated directly onto every-day garments, thus allowing for a continuous tracking of joint motions outside of a lab-constrained environment. Furthermore, when used alongside soft actuators, such as twisted coiled actuators [1], it would be possible to create a soft wearable mechatronic device that could be used during robot-assistive therapies. This would be possible because textile strain sensors are capable of reducing the overall bulkiness of the wearable robotic device by removing the need for bigger enclosures and reducing the number of wires required for communication with the mechatronic device.

The working principle of these sensors is based on measuring the change in the electrical parameters (e.g., resistance, capacitance, or inductance) of the material when it is being stretched. Based on this principle, different techniques for fabricating textile strain sensors have been proposed in the last couple of years. For example, several studies have successfully created resistive strain sensors by coating weaved or knitted textile structures with either a conductive polymer, such as the poly(3,4-ethylenedioxythiophene) (PEDOT) [2,3], graphene particles [4], or carbon nanotubes (CNT) [5], to enhance its sensing capabilities. Other studies [6,7,8] have implemented a method that consists of modifying the physical properties of a conductive fabric by laser cutting different patterns on it, and then enclosing the cut fabric inside a silicone material to create an elastic structure. On the other hand, printing techniques have also been adopted to create textile strain sensors by printing gage patterns directly onto the fabric [9,10]. Although textile strain sensors created using these methods demonstrate promising results, they can suffer from low durability due to the formation of microcracks produced after repeated strain cycles [11]. Furthermore, some of these sensors have low biocompatibility, as in the case of sensors made out of CNT, which sometimes can cause irritations when in contact with the skin for prolonged periods of time [12].

**Figure 1:**
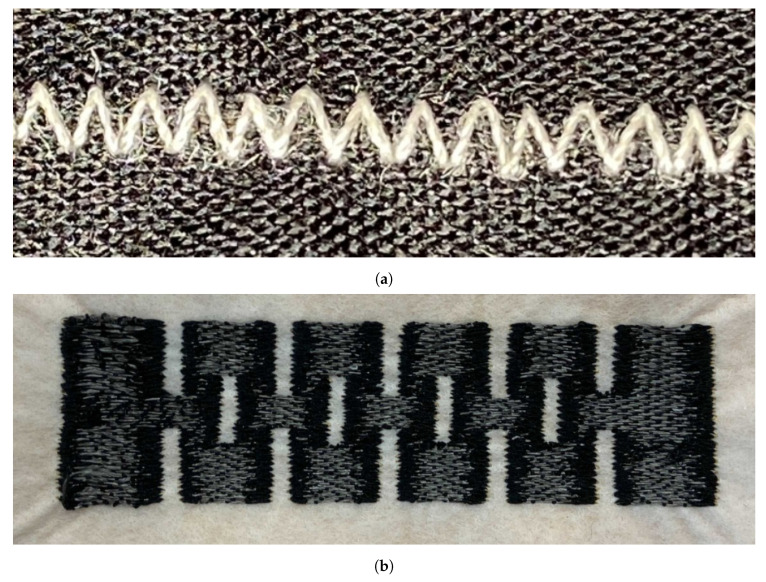
Stitching and embroidery examples. (**a**) A zigzag stitch formed by the interlock of a bobbin and needle thread. (**b**) An embroidery pattern formed by the combination of multiple stitches.

Stitching and embroidering, on the other hand, are two alternative methods used in the fabrication of textile strain sensors. Stitching can be created using three different techniques: interlooping, intralooping, and interlacing [13]. Each of these three categories can produce different classes of stitches, with the lockstitch being the most common on many commercial embroidery machines. The lockstitch stitch can be created by interlacing two threads—an upper thread and a bobbin thread—located inside an embroidery machine. An example of a lockstitch type of stitch is the zigzag stitch, as shown in Figure 1a, which is the most used in the fabrication of textile strain sensors. On the other hand, embroidering happens when multiple stitches are combined to form a specific pattern (Figure 1b). The main advantages of these two methods are that they do not require a change in the physical properties of the textile structure; instead, they use conductive threads that are directly attached to the fabric. Furthermore, the ease on the manufacturability of stitched and embroidered structures allows for a mass production of textile sensors without compromising their cost and reproducibility [14].

Although stitching and embroidering can be used to build textile strain sensors, stitching has been regarded as the preferred method of fabrication. This is because the disadvantage of embroidering is that it creates rigid structures that are not stretchable, independent of the fabric substrate onto which they are attached. Another advantage that stitching has over embroidery is that, in its relaxed state, most stitches have multiple contact points that reduce the resistance of the conductive thread used to make them. Whenever the stitches are stretched, the number of contact points between stitches decreases, which causes an increase in the resistance of the conductive thread. Several studies have taken advantage of this behaviour with good results. For example, in the study by Tangsirinaruenart and Stylios [15], different stitches were tested to create a textile resistive strain sensor. They found that the one that demonstrated the best characteristics was the zigzag stitch made out of a silver-plated conductive thread. Similarly, Park and Lee [16] studied the effect that different stitch parameters, such as stitch length, stitch shape, and stitch size, had on the performance of the strain sensor. Another study, in which a stitched sensor was implemented, was the one performed by Martínez Estrada et al. [17]. In this study, two zigzag stitches with different shapes and dimensions were overlapped on top of each other to increase the number of contact points of the conductive thread. Other studies, such as the one by Dupler and Dune [18], demonstrated that speciality stitches are more suitable for strain applications.

**Figure 2:**
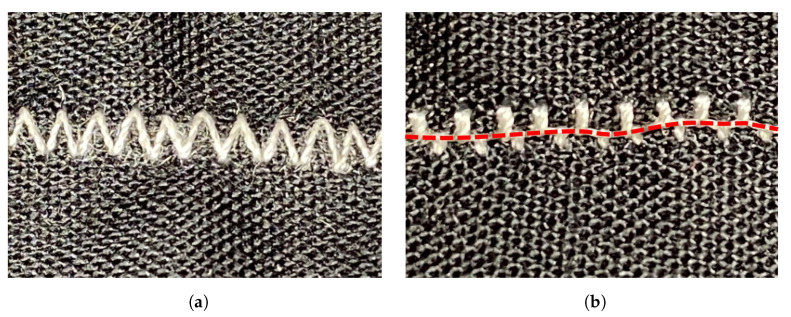
(**a**) A zigzag stitch is observed on the upper side of the fabric. (**b**) The same zigzag stitch as observed on the bottom side of the fabric. The dashed red line shows the bobbin thread.

Even when stitching presents a reliable solution for fabricating textile strain sensors, it has several inconveniences. One is that some speciality stitches require the use of technical embroidery machines that can create stitches using any of the three methods of fabrication. The other issue is that almost all stitches have different shapes on each side of the fabric, and those who do not, need to have specific parameters adjusted to maintain a consistent shape on each side. For example, a zigzag stitch will have its characteristically triangular shape on the upper side of the fabric, whereas underneath the fabric, it will look like a group of perpendicularly spaced lines if the stitch is too small (Figure 2).

This means that in almost all cases, the conductive thread should be used as the top fabric thread (the needle thread), so that there exist contact points within the stitch itself. This, however, is not always possible, as many commercial embroidery machines are not built to handle specialized threads such as metallic fibres. Unlike regular threads, conductive threads suffer from breaking and fraying due to the high friction produced as the thread goes through the many thread guides inside the machine. This issue can severely affect the performance of the strain sensor by creating discontinuous conductive paths, which result in a poor sensing ability. On the other hand, every time the conductive thread penetrates the substrate fabric, there is a chance that small fibres within the conductive thread could break and fall on the surface of the fabric. These small fibres will create conductive paths that can affect the overall performance of the strain sensor. Another problem of using the conductive thread as the top thread is that in some cases, the conductive thread can jam the embroidery machine, as this thread is thicker than regular threads. This is a major issue, as it hinders the ability of the machine to mass produce the sensors.

These problems can be avoided by using the conductive thread as the bobbin thread (on the bottom of the fabric). Moreover, by using regular embroidery machines to embroider sensors, the need for speciality stitches, that can only be made using highly specialized machines, would be avoided. However, as mentioned before, the major challenge that needs to be addressed with embroidering is creating a structure that can be stretchable, without compromising the integrity of the conductive thread. Therefore, in this study a method for building resistive textile strain sensors is presented. The main contribution of this paper is the development of a sensor fabricated using a stretchable embroidered structure, created using a commercial non-technical embroidery machine, and that shows good sensing performance. The remainder of this paper is divided as follows: Section 2 presents the methods of this study, including the design guidelines for the embroidered textile strain sensor, the data collection, and data analysis. Section 3 describes the performance metrics used to assess the sensors. Section 4 and Section 5 demonstrate the results and discussion, respectively. Finally, in Section 6, the conclusion of this study is summarized and some recommendations for future work are given.

## 2. Fabrication of Embroidered Textile Strain Sensors

**Figure 3:**
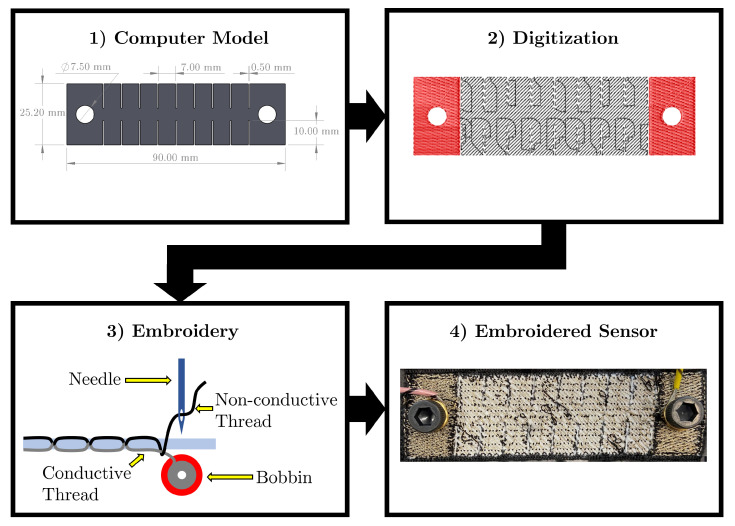
Design process of the embroidered textile strain sensor. First, a CAD model of the sensor is created. Then, the CAD model is digitized to produce an embroidery compatible file that will be read by an embroidery machine. Finally, the sensor is embroidered based on a set of specifications defined during the digitization phase.

Before embroidering, it is necessary to decide on the principle of operation of the textile strain sensor. As mentioned before, these sensors can work by measuring the changes in resistance, capacitance, or inductance produced when the sensor is stretched. In this study, it was decided that resistive sensors were the better option, as capacitance-based strain sensors can be susceptible to electromagnetic interference from anything that is conductive, such as the human body; inductive-based sensors can also be affected by other sources of noise due to their design, which resembles that of an antenna. Therefore, the proposed fabrication method for the embroidered resistive textile sensor is shown in Figure 3 and is summarized below.

### 2.1. Computer-Aided Design (CAD) Model

The first step taken in the fabrication of the embroidered textile strain sensor was to create a model of the sensor using a CAD software. The use of a CAD software is important, as it allows the sensor to be drawn with precise dimensions. One important aspect to consider during the sensor design phase is that the sensor will change its dimensions when going through the embroidery step. Depending on the embroidery setup parameters, such as the type of embroidery stitch used, the sensor length or the sensor width will be reduced depending on the stitch direction. Therefore, care should be taken when designing the sensor, as the change of dimensions can affect the sensor behaviour. This issue can be minimized by using a CAD software, as the reduction on size can be estimated and an offset can be added as part of the design.

### 2.2. Digitization

After the CAD model of the sensor is designed, it needs to be exported as a vector file, such as a drawing exchange format (DXF) file, in order to be converted into a digitized stitching pattern, which will indicate the needle paths for the embroidery machine. During the digitization step, it is important to set the appropriate parameters that will change the mechanical behaviour of the embroidered textile strain sensor. The following are the most important parameters that need to be adjusted:

- **Stitch type:** Embroidering machines are capable of creating three major types of stitches, the stroke stitch, the fill stitch, and the satin stitch. Stroke stitches can be made of running stitches and zigzag stitches and are very useful for creating lines and outlines. On the other hand, as their name implies, fill stitches are used to fill closed areas. It is important to know that fill stitches are made of multiple running stitches bundled together. Finally, satin stitches are a variation of fill stitches and are mostly used to fill small areas.
- **Stitch length:** This parameter refers to the length of each stitch. A small value will increase the total thread count of the design. For the zigzag stitch, the stitch length controls its width.
- **Stitch direction:** This option controls the direction of the stitch pattern. If the type of thread used for embroidering is not stretchable, it is important to avoid $0^{\circ }$ angles, as they will create a rigid conductive path that will break upon stretching.
- **Row spacing:** The density of the design is controlled by the row spacing. This density will affect the electrical behaviour of the sensor, i.e., the higher the density, the higher the current that will flow through the sensor [19,20]. A high density is not always desired in resistive strain sensors, as reducing the space between stitch rows may create unwanted short circuits between the conductive thread.
- **Underlay:** Enabling the underlay option will generate a series of running stitches that will secure the fabric substrate used during the embroidery process to a stabilizer substrate. Furthermore, the underlay will also prevent any distortion of the design produced by stitches pulling the fabric during the embroidery step.
- **Underpath:** This optional parameter will modify the travelling path of the stitch when moving between sections. Turning the underpath off will make the stitches run on the outline of the embroidery design. For strain sensors, enabling the underpath is preferable, as stitching around the outline of the design will prevent the embroidered structure from stretching.

### 2.3. Embroidery

After digitizing the embroidery design, the next step consists of using an embroidery machine to create the physical strain sensor. However, before embroidering, it is necessary to select the appropriate materials that will form the sensor.

#### 2.3.1. Fabric Substrate

**Figure 4:**
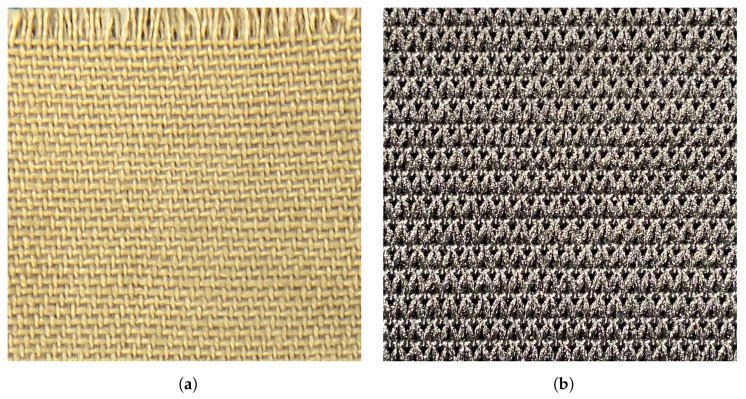
Examples of textile structures. (**a**) A close up view of a weaved structure. (**b**) A close up view of a knitted structure.

The first material that needs to be considered is the fabric substrate that will be used to attach the embroidered design. This fabric should be made out of a textile structure that allows a certain degree of stretchability without losing its original shape, i.e., the fabric should not deform after being stretched. Furthermore, if the fabric substrate presents a degree of hysteresis after being stretched, the textile strain sensor will also show this non-linear behaviour [15]. Generally, fabrics can be made by weaving or knitting multiple yarns together. A weaved fabric is constructed by interlacing yarns that are perpendicular to each other (Figure 4a), whereas a knitted fabric is made by looping together consecutive rows of yarns (Figure 4b).

While both weaving and knitting can be used as the main textile structure for the fabric substrate [21], knitted structures are preferred, given their stretchable capabilities. The amount of stretchability also depends on the type of fibres used to create the textile structure. Ideally, the fabric substrate should be made of a blend of fibres that have enough elasticity so that they can regain their original shape after being stretched. Some examples include fabrics made of polyester/spandex materials [8], polyamide fabrics combined with elastomers (Shieldex Medtex-130, V Technical Textiles Inc., Palmyra, NY, USA), polyester/elastodiene [17], and nylon/spandex [15], among others.

#### 2.3.2. Conductive Thread

The second material that needs to be selected before embroidering the textile strain sensor should be the type of conductive thread. Different types of conductive yarns exist, but the most common ones found in the development of strain sensors are metallic threads (e.g., 100% stainless steel threads), synthetic yarns coated with a fine metal layer (e.g., silver plated conductive thread), or a blended combination of metal fibres with synthetic yarn. Out of these three types of conductive threads, blended yarns are not recommended to create textile strain sensors, as the way in which the fibres within the blended yarn are arranged during its fabrication can change significantly its conductive behaviour, which can greatly affect the change in the electrical characteristics of the resulting sensor [22]. Similarly, bare metallic threads can represent a challenge, given that some of their properties may be incompatible with fabric substrates [23]. For example, dense conductive threads, such as stainless steel threads, can create a rigid structure that may not be stretchable. Instead, it is preferable to select 100% metallic threads made of very fine filaments (with diameters in the order of μm) twisted together. On the other hand, care should be taken when working with metal-coated yarns as the friction and tensile forces produced while embroidering can damage the conductive layer on the thread. This can create discontinuities in the electrical path, which can render the sensor unusable.

#### 2.3.3. Stabilizer Substrate and Embroidery Needle

Finally, the last two elements that should be taken in consideration before embroidering are the stabilizer substrate and the type of embroidering needle used. Stabilizers are another type of non-stretchable fabric that is used alongside the fabric substrate. For every embroidery design, it is important to use a stabilizer, as it will prevent the fabric substrate from moving when it is being embroidered. There are three types of stabilizer substrates: the tear-away, the cut-away, and the water soluble stabilizer. Cut-away stabilizers are not recommended for strain applications, as they are mostly used to create fixed and rigid structures. On the contrary, tear-away and water soluble stabilizers can be removed after the embroidery process is finished, with the water soluble stabilizer being the one that can be completely removed.

Regarding the embroidery needle, it is important that it is suitable for the type of thread and the type of fabric substrate used. Embroidering needles fall in two categories, ballpoint needles and cutting needles. Ballpoint needles are the preferred type when using stretchy fabrics, as their rounded tip can pierce the fabric without damaging its fibres. Cutting needles should be avoided, as they pierce the fabric by cutting its fibres. This can lead to the fabric substrate losing its stretching capabilities, and in more severe cases, create microcracks that can rip the fabric after a certain number of stretching cycles.

Another important aspect when choosing the type of needle is to select the appropriate needle size. Needle sizes are given by two numbers in the form of *Nm/S#*, where *Nm* is a metric number that represents the diameter of the needle blade in hundredths of a millimetre, and *S#* is a standard number that represents the size of the needle in the Singer (American) system. For example, a 70/10 needle indicates a needle that has a diameter of 0.7 mm, which corresponds to a number 10 needle in the Singer system. Smaller needles can damage the upper thread if the thread used is too thick, causing thread breakages. Moreover, the needle can bend, causing issues during the interlacing of the bobbing and the needle thread, which can lead to needle breakages [24].

### 2.4. Embroidered Textile Strain Sensor

After conducting some preliminary tests, the embroidered textile strain sensor was fabricated using a Janome Memorycraft 15000 automated embroidery machine that embroidered a Bekinox VN14/1x90/100Z stainless steel conductive thread (Bekaert, Zwevegem, Belgium) onto a 64%/36% polyester–rubber elastic knit structure. This knit structure was a commercially available elastic band, and its fibre composition was specified by the manufacturer. The sensor was designed in SolidWorks 2021 (Dassault Systèmes, SolidWorks Corporation, Waltham, MA, USA) using a rectangular shape, as shown in Figure 3. A series of 10 by 0.5 mm cuts were added to the rectangular shape so that the sensor could be stretched after being embroidered. Furthermore, two holes of 7.5 mm of diameter were added to each end of the design, so that they could be used to attach wires to the sensor using grommets.

**Figure 5:**
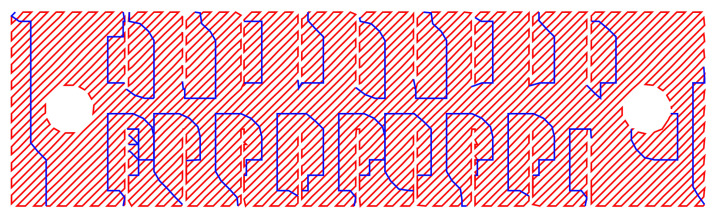
Digital representation of the embroidered textile strain sensor. The red lines represent the running stitch direction and the blue lines show the underpath that will be followed by the needle when embroidering. Changes in resistance happen when the running stitch contacts the underpath.

The resulting CAD model was further digitized using an open source scalable vector graphics editor named Inkscape with the Inkstitch extension. During digitization, a 3 mm running stitch at a $45^{\circ }$ angle with an underpath was selected to form the sensor, as shown in Figure 5.

The underpath was added because it allowed the sensor to be stretched by preventing the running stitches to run through the outline of the sensor. The second reason was to create more conductive paths when the running stitch and the underpath touched each other. Given that the Bekinox VN14/1x90/100Z stainless steel thread is made out of 90 strands of stainless steel filaments of 14 μm twisted together, the total number of strands that are in contact with the underpath changes under the applied strain. When stretched, the cross-sectional area of the conductive thread decreases, which increases the total number of contact points between the fibres that form it, therefore reducing its total resistance [25]. Furthermore, a fill stitch was used to cover each end of the strain sensor in order to attach wires for data collection. This fill stitch also prevented the sensor from being damaged after repeated cycles of stretching and unstretching.

**Figure 6:**
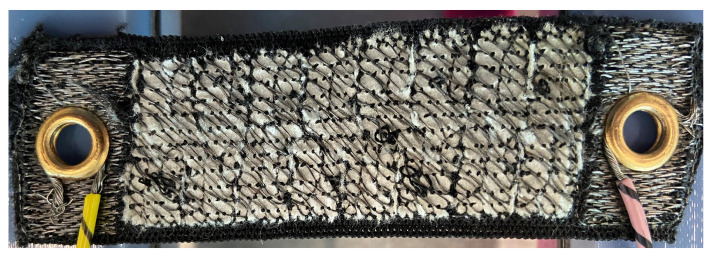
The embroidered textile strain sensor with dimensions of 90 by 25 mm. Wires are attached to each end of the sensor using grommets.

As mentioned before, the sensor was embroidered onto a polyester–rubber elastic knit fabric substrate. This type of fabric was selected as the stretchability of the knit structure combined with the rubber properties of the material, made for an elastic substrate that would regain its original shape after being stretched. Furthermore, the elastic fabric substrate was slightly stretched when placing it on the embroidery hoop; and a double layer of a tear-away stabilizer was used, as it was noted that a single layer caused irregularities in the stitches during the embroidery process. Finally, the sensor was embroidered at 400 stitches per minute (spm), which is the lowest speed that the embroidery machine used could achieve. The reason for using a low speed is to reduce the damage caused to the conductive thread during the embroidery process [26]. The completed sensor can be observed in Figure 6.

## 3. Performance Evaluation Metrics

**Figure 7:**
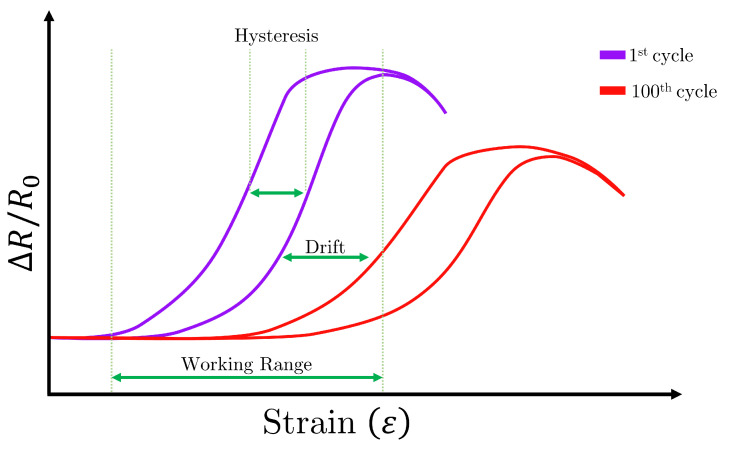
Typical response of a strain sensor when stretched. Here, several properties are shown, including hysteresis, sensor drift, and working range. Note that no scale is provided for the *x* and *y* axis, as no real data were used in this example.

In order to assess the performance of the embroidered textile strain sensor, several properties of the sensor response were measured. Some of these properties are depicted in Figure 7, and are defined as follows [15,22]:

- **Working range:** The working range is defined as the range over which the resistance changes within the range of the strain that follows a non-constant and monotonic function, i.e., the resistance increases or decreases its value as the strain changes in one direction. A sensor that increases its resistance proportionally to an increase in strain is called monotonically increasing, whereas one that decreases its resistance proportionally to an increase in strain is called monotonically decreasing.
- **Linearity:** The proportion of change in the sensor resistance with respect to the proportion of change in strain defines its linearity. The linearity of a sensor is given by the R^2^, with a sensor having an R^2^ equal to one being perfectly linear.
- **Sensitivity:** The sensitivity of a resistive strain sensor indicates the change in resistance (ΔR) with respect to an applied strain. This property is represented by the gauge factor GF and is given by the following equation:
  (1) $$GF=\frac{\Delta R/R_{0}}{\varepsilon },$$
  where ε represents the strain, which indicates a change in the sensor overall length (ΔL) under stretching conditions, and $R_{0}$ indicates the initial resistance of the sensor before stretching. The strain can be calculated as follows:
  (2) $$\varepsilon =\frac{\Delta L}{L_{0}},$$
  with $L_{0}$ indicating the sensor initial length.
- **Hysteresis:** This property refers to the difference of the sensor resistance change during an increasing and decreasing strain on any given stretching cycle. The sensor hysteresis $H_{\varepsilon }$ can be measured by finding the maximum strain difference ($\Delta \varepsilon_{h}$) between the loading and unloading cycle for a specific measured resistance value, and normalizing it with respect to the difference of the maximum ($\varepsilon_{max}$) and minimum ($\varepsilon_{min}$) applied strain, as follows [22]:
  (3) $$H_{\varepsilon }=\frac{\Delta \varepsilon_{h}}{\varepsilon_{max}-\varepsilon_{min}}.$$
- **Repeatability:** The repeatability indicates the ability of the sensor to keep its original electrical response over a certain number of cycles. When a sensor performance changes, it is said that the sensor drifts (Figure 7). Repeatability is an important parameter, as it indicates whether a sensor is reliable or not.
- **Reproducibility:** Differently from repeatability, reproducibility indicates the ability of the sensor to show the same electrical response on different sensor samples. This metric is very important, as it highlights one of the advantages of embroidery, which is the mass sensor production capabilities.

### Experimental Setup

After defining the performance metrics of the embroidered textile strain sensor, three sensor samples were created and left idled at the Wearable Biomechatronics Laboratory for one week, to condition them to the ambient temperature and relative humidity. Then, each sensor sample was tested at an ambient temperature between 22.5 and 23 °C. Data were collected using a DM3058E digital multimeter (RIGOL Technologies Inc., Portland, OR, USA). To reduce any measurement errors due to the resistance of the test probes and the sensor wires, the digital multimeter was configured to perform a 4-wire resistance, as the initial resistance of each sensor was around 26Ω. Finally, the sampling frequency of the multimeter was set to 2.5 Hz to achieve a resolution of 0.001Ω. This resolution was chosen because preliminary testing demonstrated that the change in the resistance of the sensors was around 15Ω.

**Figure 8:**
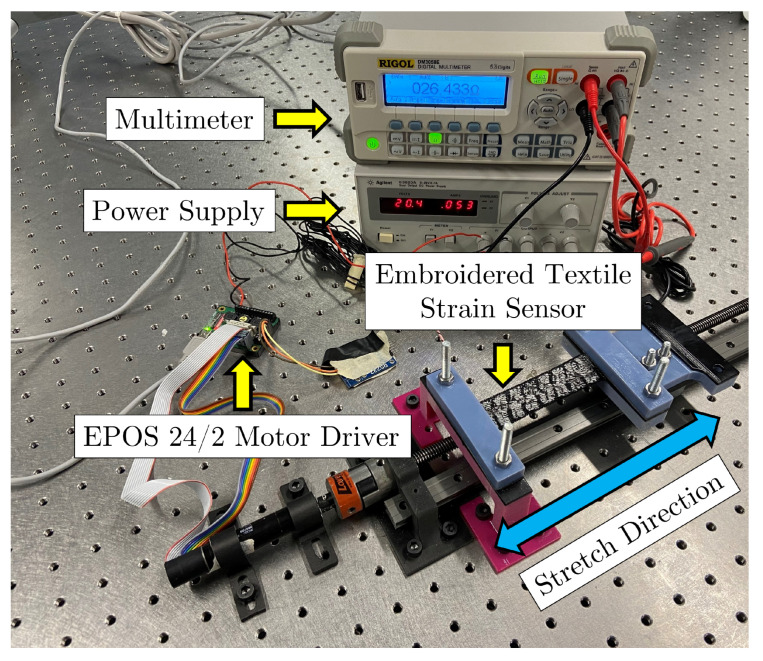
Testing setup used for collecting data from the embroidered textile strain sensors.

During the experiments, the sensors were clamped onto a moving mechanism that consisted of a lead screw attached to a motor, whose speed and position were controlled using an EPOS2 24/2 motor driver (Figure 8).

Each of the three sensors were subjected to a 66% strain at a constant speed of 69 mm/min during 100 cycles. The number of cycles was selected so that the performance of the sensors could be compared to the results obtained by Tangsirinaruenart and Stylios [15], who used the same number of stretching cycles. The speed was set to obtain as many resistance samples as possible from the multimeter, as the low change in resistance from the sensors made it difficult to obtain enough data as needed, for the further evaluation of the properties discussed in the previous section. With this speed, it was possible to obtain 218 resistance samples during each cycle, which were enough for data analysis. Finally, data from the motor driver and the digital multimeter were sent to a computer that matched the position and resistance data using a custom program written in Python [27].

## 4. Results

After testing the embroidered textile strain sensors, the collected data were post-processed and analysed offline using MATLAB R2021a (MathWorks, Inc., Natick, MA, USA). For each of the three sensor samples, the resistance data of each stretching cycle were smoothed using a 3rd order Savitzky-Golay filter with a window length of size 13. The Savitsky-Golay filter was used over other traditional smoothing methods, such as the window moving average, as it does not tend to distort the data or reduce its signal intensity [28]. The metrics discussed in Section 3 were extracted from each sensor sample.

### 4.1. Working Range

**Figure 9:**
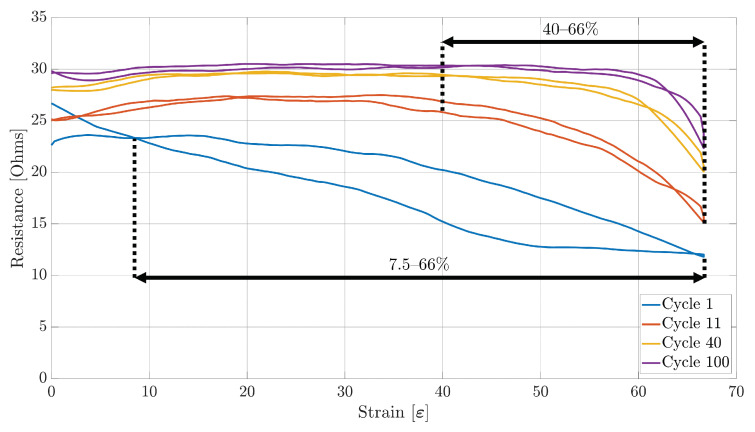
Sensor S1 strain data over the 1st, 11th, 40th, and 100th stretching cycle.

**Figure 10:**
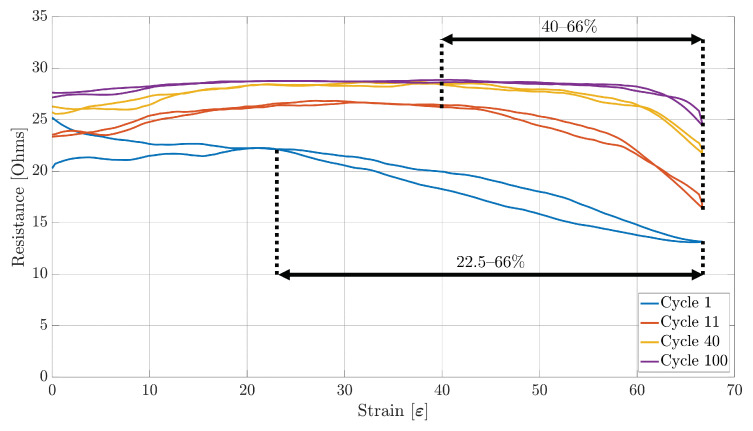
Sensor S2 strain data over the 1st, 11th, 40th, and 100th stretching cycle.

**Figure 11:**
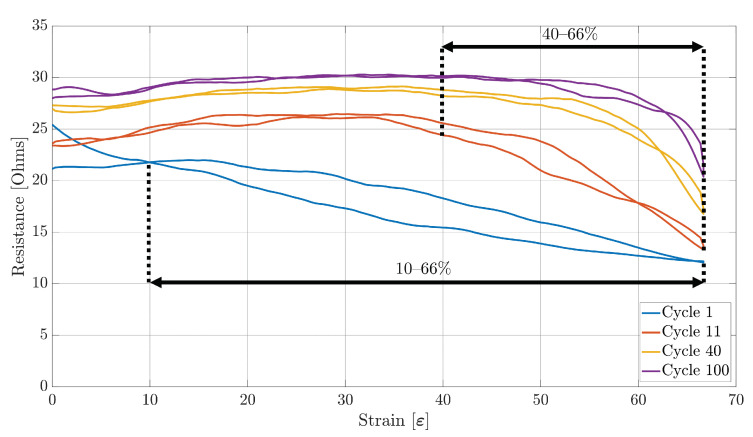
Sensor S3 strain data over the 1st, 11th, 40th, and 100th stretching cycle.

The first performance metric extracted was the working range. Figure 9, Figure 10 and Figure 11 show the plots of the change in resistance due to the strain for Sensors S1, S2, and S3, respectively, during the 1st, 11th, 40th, and 100th stretching cycle. As can be observed, the working range from each sensor decreases with each stretch cycle. For example, from Figure 9, it can be observed that Sensor S1 had an initial 7.5–66% working range. However, this working range started to decrease rapidly until the 10th cycle, when a smaller reduction on the working range was observed before stabilizing to a 40–66% range on the 40th. Although all of the three sensor samples stabilized to the same working range, Sensor S1 and S3 were the only ones that started with a working range between 7.5% and 10% of strain to a maximum of 66% of strain. On the other hand, Sensor S2 had an initial 22–66% working range (Figure 10).

Another important aspect that can be noted is that each of the three sensor samples present a decreasing monotonic behaviour during the 1st stretching cycle. However, as the stretching cycles increase, each sensor starts presenting an increasing monotonic behaviour over the range of 0–20% strain, with Sensors S2 and S3 being the ones that show a steeper increase. On the other hand, each of the three sensor samples present a non-monotonic behaviour on the range of 20–40% of strain, ending with a decreasing monotonic behaviour on the range of 40–66% of strain produced by a decrease in the resistance as the strain increases.

### 4.2. Linearity

**Figure 12:**
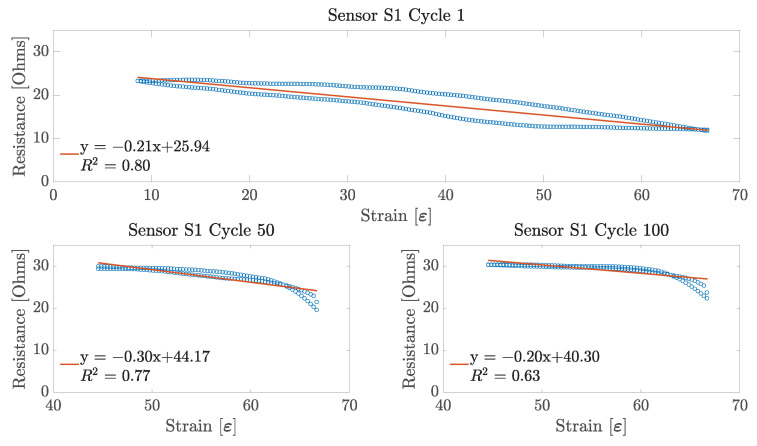
Linearity results for Sensor S1. Linearity data from Cycle 1 are shown over a 7.5–66% working range, whereas linearity data from Cycles 50 and 100 are shown over a 40–66% working range.

**Figure 13:**
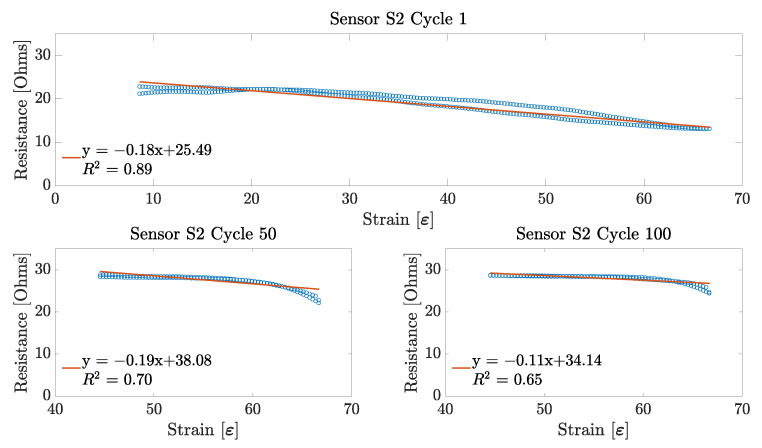
Linearity results for Sensor S2. Linearity data from Cycle 1 are shown over a 7.5–66% working range, whereas linearity data from Cycles 50 and 100 are shown over a 40–66% working range.

**Figure 14:**
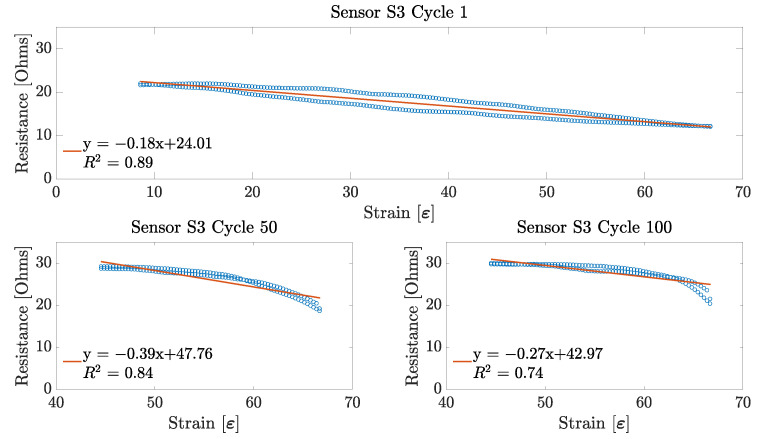
Linearity results for Sensor S3. Linearity data from Cycle 1 is shown over a 7.5–66% working range, whereas linearity data from Cycles 50 and 100 are shown over a 40–66% working range.

Having found the working range of each sensor sample, the next performance metric obtained was the sensor linearity. This parameter was obtained for each of the 100 stretching cycles across the three sensor samples. For each cycle, the linearity was obtained only for its specific working range. That is, a line of best fit was applied to a working range between 7.5% and 66% for the first 10 stretching cycles on each sensor sample; and for the remaining 90 cycles, a line was fitted to the data over the 40–66% working range for all of the three sensor samples. An example of these linear fits can be observed in Figure 12, Figure 13 and Figure 14.

From these figures, it can be observed that, for the particular case of each of the embroidered textile strain sensor, the sensors demonstrated a more linear behaviour during the first stretching cycle. This linear behaviour was consistent during the first 10 cycles for each sensor. However, as the working range of the sensor decreased, so did its linearity. Interestingly, the sensor that showed the highest linearity score during the first stretching cycle was Sensor S2, with an R^2^ equal to 0.898, followed by Sensor S3 and S1 with an R^2^ of 0.889 and 0.798, respectively. On the other hand, at the 100th stretching cycle, Sensor S3 had the highest linearity with an R^2^ equal to 0.738; and Sensors S1 and S2 had a similar low linearity with an R^2^ of 0.629 and 0.646, respectively.

### 4.3. Sensitivity and Hysteresis

With the sensors’ working range and linearity already obtained, the sensitivity and the hysteresis of each sensor during each cycle was measured. Similarly to the linearity performance, both the sensitivity and hysteresis of the sensor were obtained over the appropriate working range. From Figure 9, Figure 10 and Figure 11, it can be observed that all of the three sensors demonstrated a large hysteresis during the first stretching cycle. This behaviour varied until the 10th stretching cycle, at which point the hysteresis was around 52.11%, 70.09%, and 52.11% for Sensors S1, S2, and S3, respectively. However, starting from the 11th cycle, the total hysteresis dropped to around 7.84%, 6.46%, and 9.68% for Sensors S1, S2, and S3, respectively. These low values for each sensor remained at a similar level until the last stretching cycle.

Table 1 shows the hysteresis performance of all of the three sensor samples alongside their overall gauge factor and their linearity score. It is important to note that the hysteresis value presented in this table is the average value of the first 10 cycles, when the sensors had the highest hysteresis; and the average hysteresis percentage for the last 90 cycles, when the sensors demonstrated the lowest hysteresis score.

Furthermore, from Table 1, it can be noted that the sensitivity of the three sensors was around 1.88, as shown by the average gauge factor. Similarly to its hysteresis performance, all of the three sensor samples started with a low gauge factor, which was around 0.93, 0.8, and 0.85 for Sensors S1, S2, and S3, respectively. On the 11th stretching cycle, the gauge factor increased to 2.78, 2.43, and 3.02 for Sensors S1, S2, and S3, respectively. Unfortunately, this increase in sensitivity happened only during a few couple more cycles, as a slow decrease in sensitivity was later observed on all of the three sensors after a certain number of cycles. However, even when the sensors showed an increase and then a decrease in their sensitivity, each of the three sensors ended with an overall gauge factor that was higher in magnitude than its initial sensitivity value, as shown in Table 1.

### 4.4. Repeatability

**Figure 15:**
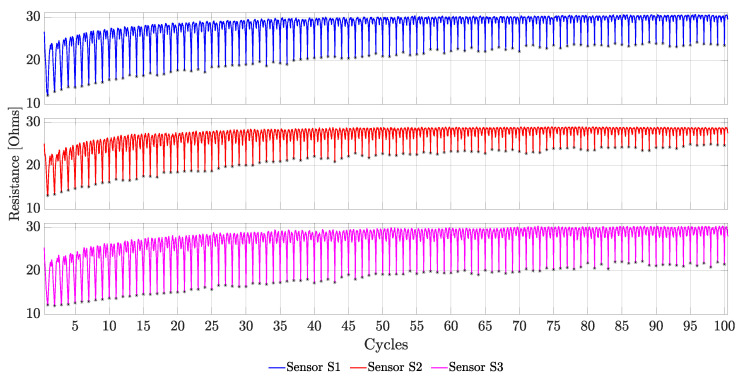
Sensor drift over the course of 100 stretching cycles. For each of the three sensor samples, the drift stabilizes around the 40th cycle. The black stars on each plot indicate the maximum strain applied (66%), which was measured halfway through the stretching cycle.

For each sensor sample, data from each stretching cycle were combined to observe the sensor repeatability performance. These data are shown in Figure 15, in which each plot represents data from each of the three sensor samples. From this figure, it can be noted that the initial resistance of each sensor on each cycle drifted upwards until it stabilized around the 40th cycle. The exception to this behaviour was during cycle number one, in which all of the three sensors demonstrated an initial mean resistance of 25.681±0.712Ω, which dropped to an average resistance of 21.463±1.129Ω on the second stretching cycle, and continued drifting upward for the rest of the 98 cycles. Similarly, an upward drift was observed for the resistance of the sensor at the maximum strain applied (66%). This drift caused a constant reduction on the working range and the sensitivity of the sensor until the 40th stretching cycle, which is when these two performance metrics stabilized. Interestingly, both Sensors S1 and S3 had a similar drift for the working range and their sensitivity across all 100 cycles. Sensor S2, on the other hand, showed a similar stabilization for its resistance value at rest with respect to the other two sensor samples. However, Sensor S2 showed a greater reduction on its resistance at the maximum strain applied compared with the other two sensors.

### 4.5. Reproducibility

Finally, the reproducibility of the embroidered textile strain sensor was assessed by finding the similarity between the data on each stretch cycle between all of the three sensor samples. This similarity test was performed using the Dynamic Time Warping (DTW) technique. DTW is a signal processing method used for aligning two time series data by non-linear mapping the data to a feature space, and then finding the optimal path (known as the warping path) that minimizes the overall cost function that compares each sample of these datasets in a one-on-one fashion [29]. The cost function will measure the distance between two points in the datasets. If the distance is small, i.e., if the two points are similar to each other, the cost function will be small. Furthermore, the similarity of the two datasets compared will be high if the cumulative cost obtained after comparing each of their data points is small.

**Figure 16:**
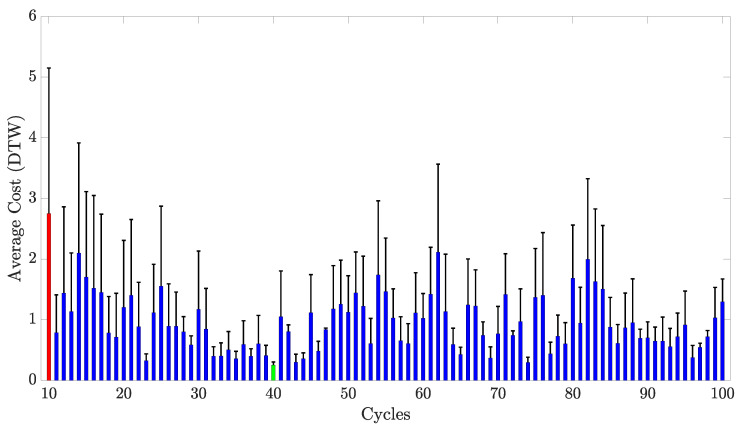
Average DTW cost across the three sensor samples for Cycles 10 to 100. The cost function was computed using the squared euclidean distance for each sensor pair combination. The highest average cost function (2.752±2.398) was for the 10th cycle (in red), whereas the lowest average cost function (0.25±0.052) corresponded to the 40th cycle and is shown in green.

Before applying the DTW technique, some data preprocessing was required. First, for each sensor sample, the resistance data collected during all the stretching and unstretching cycles were stored into a single vector to form a time series data. Then, these data were normalized using the Z-normalization, i.e., data from each sensor sample on each cycle had their mean subtracted and then divided by the standard deviation. This was performed because, as explained before, data from each sensor drifted over each cycle, creating a change in the amplitude of the signals that would have impacted the computation of the cost function on the DTW algorithm. Normalizing solves this issue by making the amplitudes of each signal similar to each other [30]. Finally, the DTW algorithm was applied to Cycles 10 to 100 over a 40–66% working range, based on the results found for the previous performance metrics. These results demonstrated that the working range of the sensor was within these limits for the last 90 stretching cycles. Moreover, the cost function used for the DTW was the squared euclidean distance, as it is the most common metric used for computing the distance between sample points in applications involving DTW. The average cost function across the 3 sensor samples for each of the 90 stretching cycles is shown in Figure 16.

Data on Figure 16 indicate that each stretch cycle was similar to its homologous cycle on a different sensor sample. With exception of the 10th cycle, the cost for all of the cycles was within the range of 0.25–2.11. Finally, the standard deviation of the average of the cost function for all cycles is also shown in Figure 16. It can be observed that similarly to the average cost, the maximum standard deviation was for the 10th cycle. On the other hand, the smallest standard deviation of the cost function happened on the 47th cycle, with a value of 0.025.

## 5. Discussion

Data presented in Table 2 show that the embroidered sensors developed in this study have a similar or improved performance in some characteristics when compared to the same characteristics presented in different studies. Note that some of the characteristics were estimated from these studies based on their presented data. To further analyse the performance of the embroidered sensor, a more detailed comparison is discussed in the following sections.

### 5.1. Working Range

**Figure 17:**
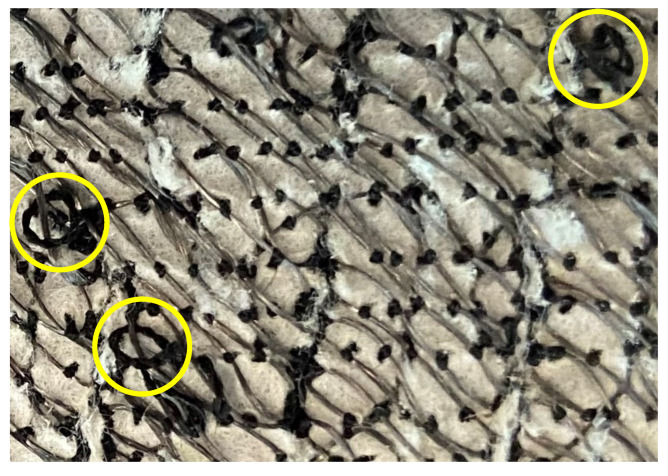
Formation of loops (circled in yellow) underneath the fabric substrate. These loops are produced due to thread tension imbalances that affect the performance of the sensor over a continuous number of stretching cycles.

As shown in Figure 12, Figure 13 and Figure 14, the working range of all three sensors decreased considerably over each stretching cycle to an overall 26%, which corresponds to the 40–66% strain. This could have happened due to changes in the lockstitch formed between the needle and the bobbing thread during the embroidery of the sensor. Initially, when the sensor has just been fabricated, the lockstich that forms the embroidery stitches is under a certain tension produced by the needle and the bobbin thread. If this tension is unbalanced due to differences in the weight between the needle and bobbin threads, or the tension parameters on the machine are not well adjusted, some looping may occur on either side of the fabric substrate. If these loops appear underneath the fabric, it is said that the bobbin thread tension is higher than the needle thread tension. When this happens, the conductive thread will move away from its initial position, which will produce an inconsistent number of contact points between the conductive thread and the underpath, or between adjacent conductive thread paths. In the case of the embroidered textile strain sensors presented in this study, small loops were observed underneath the fabric substrate, as shown in Figure 17. After stretching the sensor for the first time, the correctly formed lockstitches may have pulled the threads that were loose due to the loops formed. The change of the initial position of the thread caused a decrease in the working range of the sensor by increasing the amount of strain required to make the loose threads touch the embroidery underpath. In order to overcome this issue, slight modifications can be made during the sensor digitization phase. For example, increasing the row spacing between stitches may reduce the number of inconsistent contact points due to tension issues with the embroidery machine.

### 5.2. Linearity

Regarding the linearity of the sensors, the results presented in Section 4.2 demonstrate that the sensor is only linear during the first 10 cycles. For the remaining 90 stretch cycles, the sensor demonstrated a non-linear behaviour, as shown in Figure 12, Figure 13 and Figure 14. However, it is important to mention that all of the three sensors show a slow decrease in their resistance at the beginning of their working range (40–55% strain), which seems to be linear. Moreover, on the last part of their working range, the sensors demonstrate a steeper decrease in its overall resistance. Individually, these two changes in resistance demonstrate a linear behaviour that could be useful for applying linearizing techniques. A major issue with strain sensors is that they tend to demonstrate non-linear behaviours in the form of an exponential or logarithmic change in resistance with respect to strain, by demonstrating a non-monotonic behaviour, or simply by showing a high amount of hysteresis. However, these non-linearities can be minimized by applying deep learning techniques to create a linear model of the electrical performance of the sensor. As explained before, data from the change in resistance due to strain are a form of a time series data. This characteristic allows for the application of methods that can infer the nature of the data from past and present values. For example, in the study performed by Oldfrey et al. [31], a long short-term memory (LSTM) network was used to linearize the strain data from a stretchable conductive fabric. The results from that study demonstrated that an the LSTM network was able to accurately track the changes in resistance due to a change in the length of the conductive fabric. Similarly, in the study by Nguyen et al. [32], different linearizing methods such as LSTM networks, gated recurrent units (GRU), fully convolutional networks (FCN), and temporal convolutional networks (TCN) were tested on multiple datasets corresponding to strain sensors to compare their linearizing performance. Nguyen et al. demonstrated that both LSTM and TCN were good candidates for linearizing the strain data. This indicates that even when the embroidered sensors presented in this study showed a non-linear behaviour, it would be possible to implement the linearizing techniques discussed to improve the sensor performance.

### 5.3. Sensitivity

With respect to the sensitivity, the results demonstrated in Table 1 indicate that the sensors presented in this study demonstrate improved performance over some of the other sensors presented in the literature. For example, the sensors presented in [15] demonstrate a gauge factor between −0.0059 and 1.56 for different stitches after stretching their sensors for 99 cycles. In the case of the sensors presented here, the gauge factor varied between 1.012 (Sensor S2) and 1.713 (Sensor S3) after 99 stretching cycles. In another study [17], a gauge factor of approximately 0.5 was presented. When comparing this value to the average gauge factor of 1.491±0.465 from the sensor that had the lowest score (Sensor S2), it can be observed that Sensor S2 performs the best.

Furthermore, after observing the average gauge factor of the three embroidered strain sensors, it can be observed that its value falls somewhere in between those presented by Dupler and Dunne [18]. In their study, they created different strain sensors by stitching them at different angles on a fabric substrate. The sensors developed using that technique demonstrated an average gauge factor between −1.01 and −2.24.

### 5.4. Repeatability and Hysteresis

**Figure 18:**
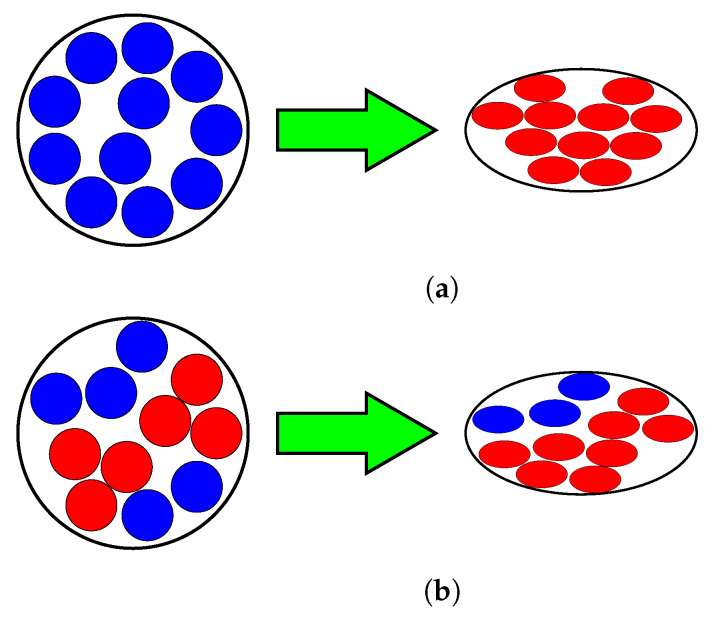
Rearrangement of fibres within the conductive thread used. (**a**) Initial position of the fibres. Each blue dot represents a fibre that is not in contact with neighbouring ones. When the sensor is stretched, the cross-sectional area of the conductive thread decreases, which increases the total number of fibres that touch each other (red dots). (**b**) Position of the fibres within the conductive thread after unstretching the sensor. Some of these fibres remain in contact with their neighbouring ones, which decreases the overall resistance of the sensor and its working range. When stretching the sensor consecutive times, some of the fibres stop contacting each other, which affects the ability of the sensor to detect changes in resistance.

In order to discuss the repeatability of the sensor, it is important to also discuss the hysteresis behaviour of the embroidered textile strain sensors. As observed from Figure 12, Figure 13 and Figure 14, a constant hysteresis decrease after cycling was observed. Table 1 shows that this decrease in hysteresis happened most noticeably after the first 10 stretching cycles. From Cycles 10 and 11, the sensors demonstrated a large reduction in their average hysteresis, which went from 42.73±16.68 to 8.54±2.66. Typically, hysteresis is produced by intrinsic properties of the material, or by the friction caused between the thread when it is stretched [22]. However, this behaviour is only common in the case when the strain sensor is made using other techniques such as knitting. In the case of the sensors presented in this study, the reduction on the hysteresis can be explained by changes in the physical characteristics of the conductive thread. The conductive thread used is made of 90 fibres of 14 μm. These fibres are plied together to form a single strand of conductive thread. When the conductive thread is subjected to a continuous mechanical motion, such as the one that happens during the stretching of the embroidered strain sensor, the fibres forming it change its initial position (Figure 18). This rearrangement of fibres creates gaps within the conductive thread that cause the overall hysteresis to decrease by preventing any further mechanical changes within the structure of the conductive thread.

As for the repeatability of the sensor, the change in position of the fibres of the conductive thread causes a drift on the performance of the sensors, as an increase in resistance can be observed. This behaviour can also be confirmed by Figure 9, Figure 10 and Figure 11, in which a rapid decrease in resistance is observed during stretching, but a slower increase in resistance happens during the unstretching phase of the sensor.

The second reason as to why the sensor drifts over time can be explained by the heating produced due to currents flowing through the sensor. Similarly to traditional strain gauges, textile strain sensors act as resistors, in which the amount of current that flows through them varies depending on the strain applied. Unfortunately, this current dissipates in the form of heat, which causes an increase in the temperature of the sensor. This increase in temperature causes the output of the sensor to vary, therefore reducing its repeatability.

Finally, the conductive thread used could have become damaged during the first stretching cycles, if the stitch direction set during the digitization step (Section 2.2) was aligned with the stretching direction. As mentioned before, this alignment should be avoided, especially if the type of stitch used is a running stitch, as being stretching in the same direction as the stitch direction may cause thread breakages.

Although the sensors demonstrate a non-repeatable behaviour, this only happens during a few initial cycle iterations. From Figure 15, it can be observed that the performance of the sensor stabilizes after the 40th stretching cycle for all of the three sensor samples. This performance remains constant for the remaining cycles, which is an indication of good repeatability. Furthermore, this behaviour shows that the sensors need to be conditioned by pre-stretching them a couple of cycles before being usable, which is similar to what other studies have found [15,33]. This pre-stretching is usually necessary because during the first few stretching cycles, some thread slippage may occur [22], the thread may suffer from creeping, or some microsnaps may be produced on the fibres that form the conductive thread, which could change its overall resistance.

### 5.5. Reproducibility

**Figure 19:**
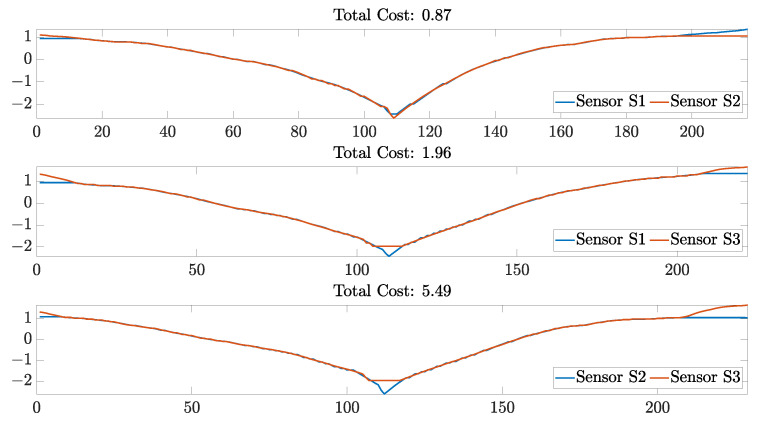
Data similarities assessed by applying the DTW technique on the resistance data of each sensor pair for Cycle 10. The *y* axis on each plot represent the normalized resistance data; and the *x* axis on each plot represents the resistance sample number compared during the computation of the warping path of the DTW algorithm.

The sensors presented in this study demonstrated consistent reproducibility between stretching cycles, as shown in Figure 16. However, it is important to address an important aspect regarding the reproducibility of the sensors. As observed on Figure 16, the standard deviations of the average cost function for some comparisons were relatively high. This was because sensor pairs had similar higher scores than others. An example of this comparison is shown in Figure 19, in which the similarity plots from the 10th cycle are shown.

**Figure 20:**
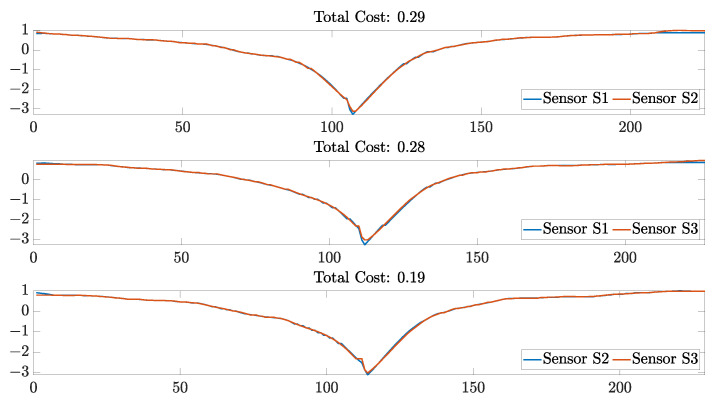
Data similarities are assessed by applying the DTW technique on the resistance data of each sensor pair for Cycle 40. The *y* axis on each plot represent the normalized resistance data; and the *x* axis on each plot represents the resistance sample number compared during the computation of the warping path of the DTW algorithm.

Data shown in Figure 19 correspond to the average cost obtained from sensor pairs that had the lowest DTW scores. It can be observed that the sensor pairs that were the most similar between each other were Sensors S1 and S2. On the other hand, sensor pairs that included Sensor S3 were the ones that had fewer similarities between each other. This could be because during the 10th cycle, specifically, Sensor S3 demonstrated a higher normalized change in resistance, which increased the distance between samples computed during the DTW algorithm. However, as the sensor response stabilized over cycles, as shown in Figure 15, the similarities between sensors increased considerably. For example, during the 40th stretching cycle, all of the sensors had the highest similarity score, with the sensor pair S2 and S3 being the most similar (Figure 20).

## 6. Conclusions and Future Work

The work presented in this study aimed towards the design of embroidered resistive textile strain sensors for use in soft robotic wearable mechatronic devices during robot-assistive therapies. The purpose of the study was to demonstrate the steps required to create a sensor that does not require a complicated fabrication procedure and that can be produced en mass. Moreover, the difference between stitching and embroidery was highlighted, and the advantages that the embroidery technique has over stitching were shown.

Furthermore, to demonstrate the feasibility of the design steps for the embroidered strain sensor, three sensor samples were created using the proposed methods, and they tested over 100 stretching cycles. In general, all of the three sensors demonstrated good sensitivity, which is comparable to recent textile strain sensors, low hysteresis behaviour, and good repeatability, as the performance of the sensor was similar after the 40th stretching cycle. However, room for improvement exists. For example, it was demonstrated that the working range of the sensors shown in this study was about 26%. As discussed by Jansen [22], the suggested working range for strain sensors used for motion tracking applications should be at least 30%. Therefore, future work should focus on increasing the working range of the embroidered textile strain sensor by tuning the embroidered parameters shown in Section 2.2, changing the overall embroidery design, or implementing a strain divider model, such as the one presented in the study performed by Basla et al. [34]. The implementation of the strain divider would be an interesting approach, as it would allow the sensor to keep the same embroidery design by adding another elastic band with a higher stiffness coefficient in parallel with the embroidered textile strain sensor. This would cause a proportional change in strain on the sensor with respect to the strain applied to the elastic band.

Regarding the repeatability of the embroidered textile strain sensor, it would be beneficial to increase the performance showed in this study. It is well known that strain sensors drift over time due to changes in the physical properties of the materials used and changes in their internal temperature. To help with this drifting behaviour, future work should focus on implementing sophisticated techniques such as transfer learning [35], to reduce changes in the electrical performance of the sensor as much as possible. Transfer learning is a useful technique, as it would allow the sensor to adapt to unknown changes by slowly shifting their data distribution until a point where this data distribution does not change significantly.

Future work should also focus on implementing the sensors created using the steps shown in this study in a sensor fusion scenario [36]. By combining the embroidered textile strain sensors with other textile sensors, such as embroidered electromyography sensors [19], it would be possible to improve the control of wearable mechatronic devices used during robot-assisted therapies. In this sense, by embedding these sensors directly on the garments, there is potential for reducing the sources of noise that traditional hard sensors present. Furthermore, embedding the sensors directly onto the garment would allow for a reduction in the number of wires required for communication, by substituting these wires with conductive thread [37,38]. However, before being able to use the textile strain sensors in a wearable mechatronic system, it would be important to properly isolate them, as sweat and other impurities can affect their correct functionality. This could be conducted by embedding the sensor within a non-conductive fabric that is also waterproof, such as neoprene. Finally, it would be beneficial to test the strain sensor over a higher number of stretching cycles (in the order of the thousands), as certain factors not mentioned in this paper, such as fatigue [33], can affect the performance of the strain sensor in the long run.

## Figures and Tables

**Table 1:** Average sensor performance metrics. Each column in the table represents a specific parameter for a sensor sample (rows) averaged across all 100 cycles (except for the hysteresis score) over its specific working range. For reference, the maximum strain applied to each sensor sample was 66%.

Sensor Sample	Linearity (R^2^)	Gauge Factor	Hysteresis (%)	
			Cycles 1–10	Cycles 11–100
Sensor S1	0.76±0.07	1.87±0.49	39.61±12.6	9.15±2.77
Sensor S2	0.74±0.09	1.49±0.47	48.15±22.01	7.99±2.79
Sensor S3	0.81±0.06	2.29±0.57	40.44±15.42	8.48±2.42
**Average**	0.77±0.07	1.88±0.51	42.73±16.68	8.54±2.66

**Table 2:** Performance comparison of stitched sensors and the embroidered sensor presented in this study.

Reference	Method of Fabrication	Stitch Type	Linearity (R^2^)	Gauge Factor	Working Range (%)	Hysteresis (%)
This study	Embroidering	N/A	0.77 ± 0.07	1.88 ± 0.51	26	8.54 ± 2.66
[15]	Stitching	Zigzag	0.98	1.61	50	6.25
		Chainstitch	0.96	3.71	25	15.1
		Overlock	0.83	0.1	16	0.98
		Coverstitch	0.97	0.21	18	2.02
[17]	Stitching	Zigzag	N/A	0.5	40	<1
[18]	Stitching	Chainstitch ^1^	0.96 ± 0.01	−1.97 ± 0.12	21	34.68 ± 3.33
		Coverstitch ^1^	0.97 ± 0.01	−1.12 ± 0.05	21	10.69 ± 4.99
		Chainstitch ^2^	0.94 ± 0.01	−2.25 ± 0.09	15	38 ± 6.18
		Coverstitch ^2^	0.93 ± 0.04	−1.01 ± 0.09	21	9.98 ± 2.14

^1^ Stitched on a 4-way knit fabric; ^2^ Stitched on a 2-way knit fabric.

## Data Availability

Not applicable.

## Abbreviations

The following abbreviations are used in this manuscript:

PEDOT	poly(3,4-ethylenedioxythiophene)
CNT	Carbon Nanotubes
CAD	Computer-Aided Design
DXF	Drawing Exchange Format
spm	Stitches Per Minute
DTW	Dynamic Time Warping
LSTM	Long Short-Term Memory
GRU	Gated Recurrent Units
FCN	Fully Convolutional Networks
TCN	Temporal Convolutional Networks
